# Further evidence of biallelic *NAV3* variants associated with recessive neurodevelopmental disorder with dysmorphism, developmental delay, intellectual disability, and behavioral abnormalities

**DOI:** 10.1007/s00439-024-02718-6

**Published:** 2024-12-21

**Authors:** Naseebullah Kakar, Selinda Mascarenhas, Asmat Ali, Azmatullah, Syed M. Ijlal Haider, Vaishnavi Ashok Badiger, Mobina Shadman Ghofrani, Nathalie Kruse, Sohana Nadeem Hashmi, Jelena Pozojevic, Saranya Balachandran, Mathias Toft, Sajid Malik, Kristian Händler, Ambrin Fatima, Zafar Iqbal, Anju Shukla, Malte Spielmann, Periyasamy Radhakrishnan

**Affiliations:** 1 Institut für Humangenetik, Universitätsklinikum Schleswig-Holstein, University of Lübeck and University of Kiel, 23562 Lübeck, Germany; 2 Department for Biotechnology, FLS&I, BUITEMS, Quetta, Pakistan; 3 Department of Medical Genetics, Kasturba Medical College, Manipal, Manipal Academy of Higher Education, Manipal, Karnataka India; 4 Department of Biological and Biomedical Science, The Aga Khan University, Stadium Road, Karachi, 78400 Pakistan; 5 Department of Zoology, Human Genetics Program, Quaid-i-Azam University, Islamabad, Pakistan; 6 Institute for Cardiogenetics, University of Lübeck, Lübeck, Germany; 7 Institute of Clinical Medicine, University of Oslo, P.O Box 1171, 0318 Oslo, Norway; 8 Department of Neurology, Oslo University Hospital, Nydalen, P.O. Box 4950, 0424 Oslo, Norway

## Abstract

Neuron navigators (NAVs) are cytoskeleton-associated proteins well known for their role in axonal guidance, neuronal migration, and neurite growth necessary for neurodevelopment. Neuron navigator 3 (NAV3) is one of the three NAV proteins highly expressed in the embryonic and adult brain. However, the role of the *NAV3* gene in human disease is not well-studied. Recently, five bi-allelic and three mono-allelic variants in *NAV3* were reported in 12 individuals from eight unrelated families with neurodevelopmental disorder (NDD). Here, we report five patients from three unrelated consanguineous families segregating autosomal recessive NDD. Patients have symptoms of dysmorphism, intellectual disability, developmental delay, and behavioral abnormalities. Exome sequencing (ES) was performed on two affected individuals from one large family, and one affected individual from each of the other two families. ES revealed two homozygous nonsense c.6325C > T; p.(Gln2109Ter) and c.6577C > T; p.(Arg2193Ter) and a homozygous splice site (c.243 + 1G > T) variants in the *NAV3* (NM_001024383.2). Analysis of single-cell sequencing datasets from embryonic and young adult human brains revealed that *NAV3* is highly expressed in the excitatory neurons, inhibitory neurons, and microglia, consistent with its role in neurodevelopment. In conclusion, in this study, we further validate biallelic protein truncating variants in *NAV3* as a cause of NDD, expanding the spectrum of pathogenic variants in this newly discovered NDD gene.

**Supplementary Information:**

The online version contains supplementary material available at 10.1007/s00439-024-02718-6.

## Introduction

Neuron navigators (NAVs), also known as navigators, are cytoskeleton-associated proteins encoded by the neuron navigators gene family. NAVs are present in most vertebrates and invertebrates. In vertebrates, this is a family of three homologs, namely NAV1, NAV2, and NAV3. In invertebrates, typically only one homolog of the NAVs is found per species; for example, the NAV3 orthologs Sick and Unc-53 in *D. melanogaster*, and *C. elegans, respectively* (Hedgecock et al. 1987; Maes et al. 2002). All NAVs, except the NAV1, contain a conserved calponin homology (CH) domain and a cytoskeletal interacting domain (CSID) at the N-terminal region, followed by two coiled-coil (CC1 and CC2) domains and an AAA + ATPase (AAA +) domain of unknown function at the C-terminal region. The CH domain contains multiple actin-binding sites through which NAVs interact with the actin cytoskeleton (Maes et al. 2002; Sandeep et al. 2023). NAVs play a key role in the development and morphogenesis of various cell types and are especially important in neuronal migration, neurite outgrowth, and overall neurodevelopment (Marzinke et al. 2013; Sánchez-Huertas et al. 2020; McNeill et al. 2011; Accogli et al. 2023).

The *NAV3* gene (MIM: *611,629), also designated as *POMFIL1* and *UNC53H*, produces alternative transcripts that encode different Neuron Navigator 3 protein isoforms. It is predominantly expressed in the developing and adult brain, with weaker expression in the adult heart, spleen, lungs, testis, and ovary (Maes et al. 2002; GTEx # phs000424.v8.p2). The expression of the *NAV3* in primary neuroblastomas has been reported to be significantly decreased suggesting its potential role in the pathogenesis of neuronal tumors as well as the nervous system development (Coy et al. 2002).

Orthologs of NAV3 in *D. melanogaster* (Sick), and *C. elegans* (Unc-53) have been reported to regulate neurite outgrowth, axon elongation, and cell migration (Hedgecock et al. 1987; Abe et al. 2014). In addition to its role in the formation of synapses at neuromuscular junctions, the role of Nav3 in heart and liver development in zebrafish has also been reported. In a study by Lv et al. (2022), *nav3* in zebrafish was found to be explicitly expressed in the heart during embryogenesis. Complete loss of *nav3* resulted in morphological and structural cardiac defects and low survival rates.

Until recently, only *NAV2* (MIM: *607,026) had been identified as a candidate gene for NDD. Biallelic truncating loss of function (LoF) variants in *NAV2* were identified in a patient with microcephaly, cerebellar dysplasia, neurodevelopmental delay, hypoplasia, and cardiac malformations (Accogli et al. 2023). Till earlier this year, this study represented the only report on the presumed loss of neuron navigator protein as the cause of NDD, thereby emphasizing the importance of understanding the role of NAVs in cellular mechanisms in central nervous system (CNS) development (Accogli et al. 2023). Earlier this year, biallelic and mono-allelic variants in also the *NAV3* gene were reported in NDD patients with intellectual disability (ID), microcephaly, and developmental delay (Ghaffar et al. 2024; Umair et al. 2024).

Here, we report loss of function variants in *NAV3* in patients consistent with dysmorphism, ID, developmental delay, and behavioral abnormalities from three independent families from South Asia and confirm that biallelic variants in *NAV3* are associated with recessive NDD.

## Materials and methods

### Families with recessive NDD studied

**Fig. 1:**
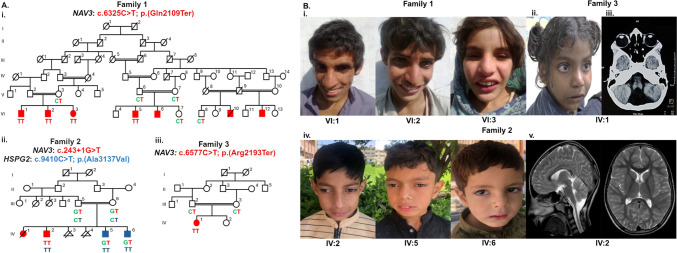
Homozygous protein truncating variants in the *NAV3* gene associated with autosomal recessive NDD. **A** Pedigree of the three unrelated families. (i) Pedigree of family 1 segregating c.6325C > T; p.Gln2109Ter variant in *NAV3*. Co-segregation of variants/genotypes is represented with: C, reference (wild type) allele; T, mutant allele. (ii) Pedigree of family 2 with *NAV3* c.243 + 1G > T and *HSPG2* variant c.9410C > T; p.Ala3137Val variants segregating independently. Co-segregation of variants/genotypes (*NAV3*) is represented with: G, reference (wild type) allele; T, mutant allele and of variants/genotypes (*HSPG2*) is represented with: C, reference (wild type) allele; T, mutant allele. (iii) Pedigree of family 3 segregating c.6577C > T; p.Arg2193Ter variant *NAV3*. Co-segregation of variants/genotypes is represented with: C, reference (wild type) allele; T, mutant allele. **B** Clinical photographs of the affected individuals. (i) VI:1, VI:2, and VI:3 of family 1, showing prominent midface, large prominent ears, and a large nose. (ii) IV:1 of family 3 showing thick and arched eyebrows, upslanted wide palpebral fissures, broad bridge of the nose, thick vermilion, wide mouth, and mild retrognathia. (iii) Brain CT of IV:1 of family 3, showing cerebellar vermis hypoplasia as represented by a yellow arrow. (iv) IV:2, IV:5, and IV:6 of family 3 show a prominent midface, large nose, and large ears, which are more pronounced in patent IV:2. (v) MRI of the brain of patient IV:2 of family 3 with no remarkable abnormality

Three unrelated consanguineous families were recruited: families 1 and 2 are from the southern and northern regions of Pakistan, respectively, and family 3 is from Manipal, India (Fig. 1A). All three families exhibited recessive NDD, consistent with dysmorphism, ID, developmental delay, and behavioral abnormalities (Fig. 1B). Written informed consent was obtained from the parents or guardians of the affected individuals. Genomic DNA was extracted from peripheral blood leukocytes using the inorganic method.

### Exome sequencing and variant analysis

Briefly, ~ 1000-ng total DNA from the two affected individuals (VI:2 and VI:5) in family 1 was used for hybridization-based exome enrichment. The Illumina DNA Prep with Exome 2.0 Plus Enrichment Kit (Illumina, San Diego, CA, USA) was used for the exome target capture. Sequencing was performed on the Next-seq 2000 (Illumina, San Diego, CA, USA). Hybridization-based exome enrichment by Agilent SureSelect human (all Exome) V6 (Agilent Technologies, Santa Clara, CA, USA) was performed for the DNA samples from the two affected individuals (IV:2 and IV:5) in family 2. Sequencing was performed on the Illumina platform, NovaSeq 6000 (Illumina, Santa Clara, CA, USA). In family 3, Singleton exome sequencing (ES) by massively parallel sequencing on the Illumina platform (Illumina, San Diego, California, USA) using the TWIST Bioscience capture kit was done for the proband (IV:1). Alignment of the cleaned reads to the human reference genome (GRCh38/hg38), variant calling, and annotation were performed as described previously (Ullah et al. 2019; Kakar et al. 2024; Yousaf et al. 2022, 2023; Kausthubham et al. 2021).

### Variant analysis

Variant analysis of family 1 was performed using the VarFish pipeline v0.23.9 (Holtgrewe et al. 2020). The filters were set to prioritize variants based on the following criteria: detection of nonsynonymous, indels, and splice sites (± 12 bp) variants, exclusion of variants with minor allele frequency (MAF) > 0.001 in the genome aggregation database (gnomAD v4.0) (Koenig et al. 2024) and detection of homozygous variants shared between the two affected individuals in family 1 as an autosomal recessive model. Furthermore, the variants filtered based on the criteria mentioned above were prioritized according to bioinformatics predictions of their impact, as determined by the Combined Annotation-Dependent Depletion (CADD) tool (Kircher et al. 2014; Rentzsch et al. 2019) and phenotype analysis based on the Human Phenotype Ontology (HPO) approach (Köhler et al. 2014). Additionally, an online tool “AutoMap” was used for the detection of regions of homozygosity (RoH) (Quinodoz et al. 2021).

Variant analyses of families 2 and 3, was performed using the WES analysis pipelines described previously (Yousaf et al. 2022, 2023; Molina-Ramírez et al. 2022; Kausthubham et al. 2021).

Verification and segregation analyses of the prioritized variants were performed using Sanger sequencing. The nomenclature of the variants identified in *NAV3* is based on the transcript NM_001024383.2.

### Homozygosity mapping

The VCF files of probands were used by the AutoMap (Quinodoz et al. 2021) tool (v1.0), to determine the region of homozygosity (ROH).

### Expression of *NAV3* in the embryonic and adult human brain

The single-cell RNA transcriptomes dataset of developing human brain tissue, generated from 10 distinct brain regions from 13 individuals, as reported in a recent publication by Bhaduri et al. (2021), was accessed and downloaded from the UCSC cell browser (Speir et al. 2021). This dataset was used to analyze the expression pattern of *NAV3* in the developing human brain from (time points) gestation week (GW)14 to GW25.

Similarly, the single-cell RNA transcriptomes dataset of the young adult human brain, generated by Velmeshev et al. (2019) was used for the expression analysis of *NAV3*. The expression of *NAV3* in the young adult human brain from (time points) 4 years to 20 years was analyzed.

The filtered raw expression count matrices, metadata files, and UMAP/TSNE embeddings were used to construct Seurat (v5) objects. Feature plot function was used to plot the raw expression values of *NAV3* in the UMAP embedding provided in the original publication, where original cell type annotations were used. For visualizing the *NAV3* expression in violin plots, the expression was log-normalized using the Normalize Data function, and all the zero-counts were removed.

## Results

### Clinical features of affected individuals

The main clinical features of the affected individuals from the three unrelated families are summarized in Table 1. Affected individuals presented with dysmorphism, ID, developmental delay, and behavioral abnormalities.

**Table 1:** Clinical features observed in individuals with *NAV3*-related neurodevelopmental disorder

Study	Our study					Ghaffar et al. (2024)	Umair et al. (2024)
Family ID	F1			F2*	F3	n = 7	F11
Patient ID	VI:1	VI:2	VI:3	VI:2	IV:1	n = 11	IV:4
Gender	Male	Male	Female	Male	Female	Males = 6/11 Female = 5/11	Female
Ethnicity	Pakistani			Pakistani	Indian	Pakistani = 3/6 Caucasian = 1/6 European = 2/6	Saudi Arabian
Consanguinity	Consanguineous			Consanguineous	Consanguineous	Consanguineous = 3/7 Non- consanguineous = 4/7	Consanguineous
Variant in *NAV3* (NM_001024383.2)	c.6325C > T; p.(Gln2109Ter)			c.243 + 1G > T; p?	c.6577C > T; p.(Arg2193Ter)	Missense = 3/7 Stop gain = 2/7 Frameshift deletion = 1/7 Frameshift insertion = 1/7	c.2604_2605delAG;p.(Val870SerfsTer12)
Zygosity	Homozygous			Homozygous	Homozygous	Homozygous = 4/7 De novo heterozygous = 2/7 Heterozygous inherited = 1/7	Homozygous
Age last evaluated	33 years	22 years	20 years	14 years	5 years	04–50 years	13 years
Age of onset	By birth	By birth	By birth	By birth	9 months	By birth—14 years	9 months
Clinical findings							
Developmental delay	Yes	Yes	Yes	Yes	Yes	6/6	Yes
Intellectual disability (degree)	Mild	Moderate	Moderate	Mild	Mild	Mild = 6/11 Moderate = 2/11 Severe = 3/11	Mild
Microcephaly	Yes	Yes	Yes	Yes	No	Yes = 5/10 No = 5/10	NA
Dysmorphism	Yes	Yes	Yes	Yes	Yes	Yes = 5/11 No = 6/11	Yes
Seizures	No	No	No	No	No	NA	Yes
Tone	No	No	No	Yes (generalized hypotonia)	NA	Hypotonia = 3/3	Generalized hypotonia
Behavioral abnormalities	Yes	Yes	Yes	Yes	Yes	Yes = 7/10 No = 3/10	No

*NA* not available, *CT* computed tomography, *MRI* magnetic resonance imaging

^*^Individuals IV:2, IV:5 and IV:6 in family 2 had a homozygous variant, c.9410C > T; p.(Ala3137Val) in the *HSPG2* (NM_005529). IV:2 were homozygous whereas IV:5 and IV:6 were carriers of the NAV3 variant

### Family 1

Family 1 is a large consanguineous family originating from southern Pakistan and segregates NDD with an autosomal recessive mode of inheritance (Fig. 1A-i). In this family, NDD was present in 7 affected individuals (6 males and 1 female) in three nuclear families, including one deceased individual (VI:10) due to unknown reasons (Fig. 1A-i). Affected individuals presented with dysmorphism, microcephaly, ID, and developmental delay (Fig. 1B-i).

At the time of physical assessment, detailed clinical features were obtained for three affected individuals: VI:1 age 33 years, VI:2 age 22 years, and VI:3 age 20 years (Fig. 1B-i). Their occipitofrontal circumferences (OFC) measurements were 53 cm (1.42 SD), 52 cm (−2.09 SD), and 52 cm (−2.17 SD), respectively, consistent with mild microcephaly (Supplementary Table 1). These three individuals had mild to moderate ID and developmental delay. Dysmorphic features included a prominent midface, apparently large prominent ears, and a large nose (Fig. 1B-i). They had normal height, absent speech, no cleft lip or cleft palate, no seizures, and no single central maxillary incisor was observed. Additionally, they exhibited behavioral abnormalities such as hyperactivity, aggressiveness, and attention deficit. They showed neither a social smile nor cooing sounds (Table 1 and Supplementary Table 1). Echocardiography was performed for VI:2, which revealed an apparently normal heart.

### Family 2

The family 2 with complex neurological and developmental features was ascertained from northern Pakistan (Fig. 1A-ii). The three affected brothers (IV:2, IV:5, and IV:6) and a deceased girl (IV:1) presented with recessive NDD (Table 1 and Supplementary Table 3). The clinical features common among the three patients are dysmorphism, mild to moderate ID, developmental delay, and early-onset nystagmus.

The dysmorphic features include a prominent midface, apparently large prominent ears, and a large nose which was more prominent in IV:2 (Fig. 1B-iv). Remarkably, IV:2 had microcephaly with an occipitofrontal circumference of 49 cm (−3.5 SD). They had social smile, normal cooing sounds, normal height, normal speech, no cleft lip or cleft palate, and no seizures.

### Family 3

Proband IV:1 in family 3 from Manipal, India was ascertained at five years of age (Fig. 1A-iii). She was born to a consanguineously married couple at term. Her antenatal history was uneventful, and her birth weight was 2.5 kg (−1.76 SD). Behavioral abnormalities noted were aggressiveness, hyperactivity, lack of eye contact, and non-verbal communication. She was noted to have delays in motor milestones. Neck holding was attained at one year, rolling over at two years, crawling after two years, sitting without support at three years, standing after three years, and walking at five years of age. Language milestones were also grossly delayed; she could only speak bi-syllables by the age of five years.

On examination at five years of age, her height was 116 cm (−1.2 SD) and her head circumference was 48 cm (−1.33 SD). She was noted to have dysmorphic features such as thick and arched eyebrows, up-slanted and wide palpebral fissures, broad bridge of the nose, thick vermilion, wide mouth, and mild retrognathia (Fig. 1B-ii). The ophthalmologic evaluation carried out was unremarkable. Computed tomography of the brain showed cerebellar vermis hypoplasia (Fig. 1B-iii).

### Exome sequencing identifies homozygous nonsense and splice site variants in *NAV3*

To identify the pathogenic variant, two affected individuals (IV:2 and VI:5), one from each of the two branches of the pedigree of family 1, were selected for exome sequencing (Fig. 1A-i). As the disease segregating in family 1 is rare and transmits in an autosomal recessive mode of inheritance, we applied a filtration strategy as described in methods to detect rare homozygous variants. Further ranking of the filtered variants based on HPO term for intellectual disability (HP:0010864) and CADD score > 20, identified a homozygous nonsense variant c.6325C > T; p.(Gln2109Ter) in the *NAV3* (NM_001024383.2) gene. This variant was found in a large homozygous region of 23.4 Mb (Chr12:76,386,271–99,825,429) shared by both the affected individuals (VI:2 and VI:5), as determined by AutoMap from their exome data (Supplementary Table 2 and Supplementary Fig. 2A-i). *NAV3* appeared to be the best candidate after excluding the benign or likely benign variants in other genes.

Notably, *NAV3* is predicted to be intolerant to loss of function variants, with a pLI score of 1 (gnomAD v4.0 project; Karczewski et al. 2019). The identified nonsense variant p.(Gln2109Ter) is predicted to create a premature stop codon and a truncated NAV3 protein with 2109 amino acids compared to wild-type protein with 2385 amino acids. The variant is ultra-rare with MAF: 1/ 1,432,136 in gnomAD v4.0. The variant is predicted to be pathogenic by several bioinformatics tools, including CADD, with a CADD score of 46 (Table 2). Finally, we confirmed by Sanger sequencing that the variant segregated in the extended family (Fig. 1A-i and Supplementary Fig. 1).

**Table 2:** Variants identified in *NAV3* in families with recessive NDD

Family	Genomic position	Zygosity	Variant type	Gene	Variant		Bioinformatic analysis				MAF	ACMG
					nc Change	aa Change	ClinPred	Splice AI score	CADD Score	phyloP100	gnomAD v4.0	
Family 1	chr12:78,197,280	Homozygous	nonsense	*NAV3*	c.6325C > T	p.Gln2109Ter	1	n.a	46	7.91	1/ 1,432,136	Pathogenic PM2, PM2, PP1 and PM4
Family 2*	chr12:77,831,705	Homozygous	splice site	*NAV3*	c.243 + 1G > T	p.??	n.a	0.99	33	7.99	2/1,597,914	likely pathogenic PP1,PM2 and PP3
	chr1:21,841,204	Homozygous	missense	*HSPG2*	c.9410C > T	p.Ala3137Val	0.16	n.a	19	1.7	3/1,613,864	VUS PM2, PP3, PP1 and BP4
Family 3	chr12:78,199,393	Homozygous	nonsense	NAV3	c.6577C > T	p.Arg2193Ter	1	n.a	44	2.67	2/1,610,416	Pathogenic PP1, PM2, PP3 and PM4

Genomic positions are according to GRCh38/hg38 human genome assembly

*RoH* region of homozygosity, *Mb* megabase, *Chr* chromosome, *aa* amino acid, *MAF* minor allele frequency, *VUS* variant of uncertain significance

^*^Individuals IV:2, IV:5 and IV:6 in family 2 had a homozygous variant, c.9410C > T; p.(Ala3137Val) in the *HSPG2* (NM_005529). IV:2 were homozygous whereas IV:5 and IV:6 were carriers of the *NAV3* variant

In family 2, ES performed on individual IV:2 (Fig. 1A-ii) identified a rare (MAF: 2/1,597,914 in gnomAD v4.0) homozygous variant c.243 + 1G > T in the *NAV3* gene. The variant was predicted to be pathogenic (CADD score 35) and to alter the splice donor site of exon 1, resulting in aberrant splicing (Splice AI: 0.99) (Table 2). Validation of the variant c.243 + 1G > T followed by segregation analysis by Sanger sequencing revealed that the variant was homozygous in the affected individual (IV:2) and heterozygous in his unaffected parents. However, the variant was also heterozygous in both the affected siblings IV:5 and IV:6 (Fig. 1A-ii and Supplementary Fig. 1). Since the variant is ultra rare and predicted to have a strong effect, resampling and repetition of segregation analysis were performed to exclude the sample mix-up or any technical error. However, the results confirmed the earlier segregation analysis, revealing that both affected siblings IV:5 and IV:6 were indeed heterozygous for the *NAV3* variant. ES performed for IV:5 (Fig. 1A-ii) heterozygous for the *NAV3* variant, revealed a missense variant c.9410C > T; p.(Ala3137Val) in the *HSPG2* gene (NM_005529) (Table 2). The missense variant is rare in gnomAD v4.0 (MAF: 3/1,613,864) and segregates in the extended family (Fig. 1A-ii). Bioinformatics analysis using CADD predicts this variant to be of uncertain significance (VUS). Altogether, it can be concluded that both variants in *NAV3* and *HSPG2* segregate independently in family 2 (Fig. 1A-ii). This finding is consistent with the autozygosity analysis by the AutoMap tool. Specifically, the *NAV3* variant in patient IV:2 is within a 18.6 Mb RoH absent in patient IV:5, who is homozygous for the *HSPG2* variant. Additionally, the *HSPG2* variant is within a 6.8 Mb RoH shared by patients IV:2 and IV:5, both of whom are homozygous for *HSPG2* and heterozygous for the *NAV3* variant (Supplementary Fig. 2A-ii and B).

In family 3, ES analysis of IV:1 (Fig. 1A-iii), identified a homozygous variant, c.6577C > T; p.Arg2193Ter in exon 37 of *NAV3* gene (NM_001024383.2). Sanger validation and segregation of the variant confirmed the carrier status in her parents (Supplementary Fig. 1). This variant is rare with a MAF of 2/1,610,416 in the gnomAD v4.1.0 database. This variant is also absent in the in-house database of 3200 local exome datasets. The variant p.(Arg2193Ter) was predicted to be pathogenic (CADD score: 44) and to create a premature stop codon, resulting in a truncated NAV3 protein with 2192 amino acids compared to wild-type protein with 2385 amino acids. Consistent with family 1, the variant was found in a large homozygous region of 64.7 Mb (chr12:52,451,817–117,222,662) as identified by AutoMap (Supplementary Fig. 2A-iii).

Overall, these results confirm that protein truncating variants in *NAV3* are likely a rare cause of autosomal recessive NDD.

### Expression of *NAV3* in the embryonic and adult human brain

*NAV3*, among other tissues, is strongly expressed in the brain in mice and humans. However, its specific expression pattern in the developing and adult human brain is unclear. We re-analyzed published single-cell transcriptomic datasets of the human embryonic (GW14 to GW25) whole brain and young adult (4 to 20 years) human cortex (Fig. 3A–D). The analysis revealed that *NAV3* is expressed across various cell types in embryonic and adult human brains. In general, the percentage of cells that express *NAV3* is higher in young adult brains than in embryonic brains across all cell types, with a higher expression in early and late-born excitatory neurons, caudal and medial ganglionic eminences (CGE/MGE) derived inhibitory neurons, and microglia.

In both datasets, the expression is the highest in several neuronal cell types as well as microglia. In the embryonic brain, more than 12% of both excitatory and inhibitory neurons express *NAV3* (Supplementary Fig. 3B). In the cortical tissues of young adult brains, more than 87% of the neurons of the cortical layers express *NAV3*. In both these datasets, the *NAV3* expression is generally reduced in non-neuronal cells, except for microglia (Fig. 3A-D). This finding further supports the role of NAV3 in axonal guidance and cell migration during early neurodevelopment.

## Discussion

We report five individuals from three unrelated families with biallelic variants in *NAV3* with variable dysmorphic features, developmental delay, mild to moderate ID, and behavioral abnormalities. The clinical features of the individuals in this study and previously reported individuals are summarized in Table 1 (detailed findings are available in Supplementary Table 1). The role of NAV3 in the nervous system and its association with ID and NDD has not been extensively investigated. However, in an exome-wide association study (ExWAS) of a large cohort of patients with autism spectrum disorder (ASD), six genes including *NAV3* were significantly associated with ASD as a moderate-risk gene. The association of *NAV3* with ASD as a moderate-risk gene was primarily based on rare loss of function (LoF) variants with ExWAS significance (Zhou et al. 2022). Recently, Ghaffar et al. reported for the first time biallelic and mono-allelic variants in the *NAV3* in 11 individuals from seven unrelated families (Ghaffar et al. 2024). Clinical findings such as developmental delay, mild to severe ID, microcephaly, behavioral abnormalities like hyperactivity and aggression, and variable dysmorphic features were noted in these individuals. Other findings included hypotonia, dystonia, and pigmentary skin changes. MRI scans of the brain was available for one individual, which revealed mild delayed myelination. The individual IV:2 from family 3 of our study had CT brain findings of cerebellar vermis hypoplasia. Later, Umair et al. reported a 13-year-old female with a biallelic frameshift deletion in *NAV3,* who presented with developmental delay, mild ID, and hypotonia, with normal brain MRI (Umair et al. 2024). Based on the phenotypes of previously reported individuals, the variants we identify can be interpreted to be the likely cause of the phenotypes observed in our study.

**Fig. 2:**
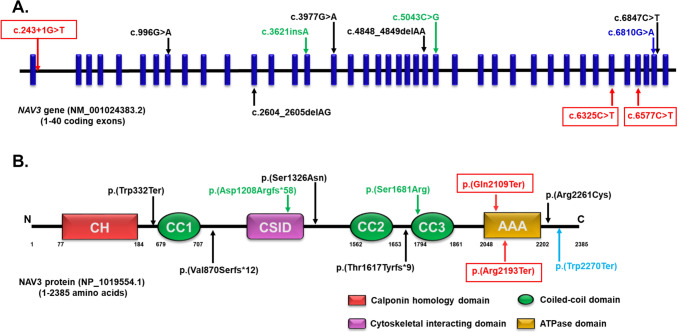
Schematic representation of human NAV3 gene and protein with functional domains. **A** Schematic representation of reported variants in *NAV3* gene. **B** and its position in the NAV3 protein domain. The homozygous variants in black, heterozygous de novo variants are shown in green and heterozygous inherited variants in blue. The variants reported in our study are shown in red. *N* N-terminal, *C* C-terminal, *CH* calponin homology domain, *CSID* cytoskeletal interacting domain, *CC* coiled coil domain, *AAA* ATPase domain

*NAV3* is located at 12q21.2 and contains encodes for a cytoskeletal-associated protein NAV3 of 2385 amino acids. To date, five biallelic and three mono-allelic variants including missense, and frameshift insertion/deletions have been reported in 12 individuals from eight unrelated families with NDD (Figs. 2A and B). In addition, 12 frameshift, nine nonsense, and three splice site *de* novo variants were reported in the ASD cohort of the ExWAS study. In our study, we report two homozygous nonsense and a splice site variant in three unrelated families. The nonsense variant p.(Gln2109Ter) identified in family 1 and the p.(Arg2193Ter) in family 3 are situated in the ATPase domain of the protein and are predicted to result in protein truncation (Fig. 2A and B). The third variant, c.243 + 1G > T, identified in family 2, affects a highly conserved guanine nucleotide (PyloP100: 7.99) predicted to lead to aberrant splicing. Thus, these three variants may result in the formation of a truncated NAV3 protein, or the transcript may undergo nonsense-mediated mRNA decay thereby leading to loss of *NAV3* function. Heterogeneous clinical manifestations, as well as both autosomal dominant and recessive patterns of inheritance, were observed in individuals from these three families with *NAV3* variants. Hence establishing a genotype–phenotype correlation becomes challenging.

In family 2, the patient harboring a homozygous *NAV3* variant (c.243 + 1G > T) presents with the most notable clinical manifestations of microcephaly and learning disabilities. Neuroimaging assessment of this individual reveals relative atrophy of the cerebral peduncles and a flattened appearance of the spinal cord in the visualized segment. In contrast, the MRI findings for the sibling (IV:5) carrying a heterozygous *NAV3* variant appear unremarkable. Additionally, the proband (IV:2) and other affected siblings (IV:5 and IV:6) exhibit a spectrum of developmental delay, intellectual disabilities, behavioral abnormalities, and early-onset nystagmus (Supplementary Table 3).

These collective observations suggest that the homozygous *NAV3* variant may be associated with a mild phenotypic presentation in the proband (IV:2). We hypothesize that the potential co-occurrence of an additional rare variant in the *HSPG2* could contribute to the observed phenotypic variability within family 2 (Fig. 1A-ii).

Earlier studies investigating NAV3 orthologs in *Drosophila* (Sick) have demonstrated their role in neurodevelopment and neurogenesis (Hedgecock et al. 1987; Abe et al. 2014). Sick is the only ortholog of the three NAV (NAV1-3) proteins. The Sick protein in *Drosophila* shares approximately 40% amino acid identity with human NAV1, 56% with NAV2, and 52% with NAV3. A recent study by Accogli et al. (2023), investigated the role of Sick in *Drosophila*, a homolog of NAV3. Sick was found to be strongly expressed in the brain of *Drosophila*, and its mutants were mostly lethal or exhibited neurobehavioral phenotypes (Accogli et al. 2023). Additionally, overexpression of pathogenic NAV3 variants in HEK293T and COS7 cells was found to destabilize microtubules (Ghaffar et al. 2024). Further, the role of NAV3 in neurodevelopment was assessed by morpholino antisense oligonucleotides (MO) that interfere with the translation of *Nav3* in zebrafish. The *nav3* morphants (*nav3*-MO) were found to have severe behavioral and morphological defects, including microcephaly and impaired neuronal growth. These phenotypes were rescued with human wild-type *NAV3* mRNA, indicating the role of NAV3 in neurodevelopment, which if impaired leads to NDD (Ghaffar et al. 2024).

**Fig. 3:**
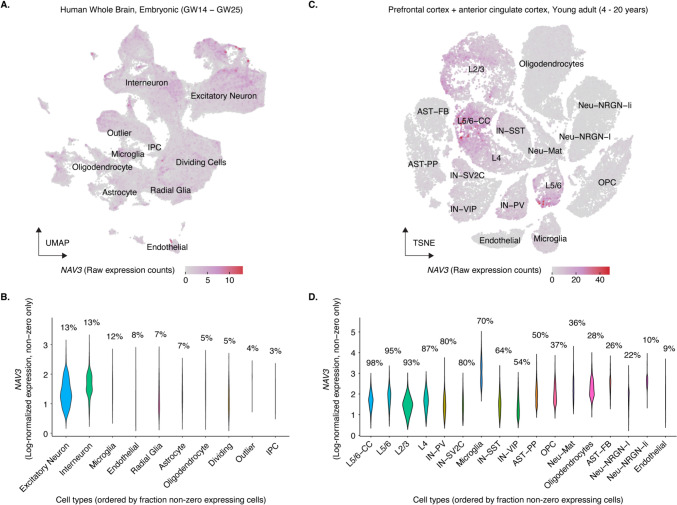
Expression of *NAV3* in human whole embryonic brain and prefrontal and anterior cingulate cortex of young adult. **A** embryonic human brain (GW14–GW25) and raw expression values in the original UMAP/TSNE embeddings provided by the respective studies (Bhaduri et al. 2021). **B** young adult human prefrontal cortex and anterior cingulate cortex (4–20 years) and raw expression values in the original UMAP/TSNE embeddings provided by the respective studies Velmeshev et al. (2019). **C**–**D** Distribution of non-zero log-normalized expression values, across clusters. The percentages above each violin refer to the fraction of cells per cell type that express *NAV3* (i.e., non-zero expression). Cell types are based on the annotations in the original publication

Furthermore, in our re-analysis of scRNA transcriptomic datasets of the embryonic and young adult human brains from the previously published papers (Bhaduri et al. 2021; Velmeshev et al. 2019), we found strong expression of *NAV3* in neuronal cell types, especially in early and late-born excitatory neurons, showing the important role of NAV3 in neurodevelopment (Fig. 3A–D).

In summary, our findings provide further evidence of biallelic variants in *NAV3* as the cause of recessive NDD and expand the allelic and phenotypic spectrum of NDD caused by homozygous *NAV3* variants.

## Supplementary Information

Below is the link to the electronic supplementary material.

Supplementary file1 (TIFF 9486 KB) SUPPLEMENTARY FIGURE 1. Part of NAV3 Sequence chromatogram i) unaffected father (V:6) and a patient (VI:5) of family 1, showing the c.6325C>T; p.(Gln2109Ter) variant, ii) unaffected mother (III:4) and a patient (VI:2) of family 2, showing the c.243+1G>T variant. iii) unaffected father (III:2) and a patient (VI:1) of family 3, showing the c.6577C>T p.(Arg2193Ter) variant

Supplementary file2 (TIFF 13713 KB) SUPPLEMENTARY FIGURE 2. Regions of homozygosity (RoH). A) RoH region containing NAV3 by AutoMap i) a 23.4 Mb region on chromosome 12 (Chr12:76386271-99825429) (encircled by red dotted lines), containing NAV3 gene, is shared by both the affected individuals (IV:2 and VI:5) in family 1. ii) an 18.6 Mb region on chromosome 12 (chr12:71,003,360-89,624,902), containing NAV3 by affected individual IV:2 in family 2. iii) a 64.7 Mb region on chromosome 12 (chr12:52,451,817-117,222,662), containing NAV3 by affected individual IV:1 in family 3. B) ROH region containing HSPG2 by AutoMap i) a 6.80 Mb region on chromosome 1 (chr1:17,275,669-24,083,649) containing HSPG2 gene, is shared by the affected individuals (IV:2 and VI:5) in family 2

Supplementary file3 (XLSX 21 KB)

Supplementary file4 (XLSX 11 KB)

Supplementary file5 (XLSX 12 KB)

## Data Availability

No dataset was generated during the current study. The dataset analyzed for scRNA seq is available online at https://dev-brain-regions.cells.ucsc.edu for the whole embryonic brain and for prefrontal and anterior cingulate cortex of young adults at https://autism.cells.ucsc.edu
